# Using the longest significance run to estimate region-specific p-values in genetic association mapping studies

**DOI:** 10.1186/1471-2105-9-246

**Published:** 2008-05-27

**Authors:** Ie-Bin Lian, Yi-Hsien Lin, Ying-Chao Lin, Hsin-Chou Yang, Chee-Jang Chang, Cathy SJ Fann

**Affiliations:** 1 Department of Mathematics, National Changhua University of Education, Changhua 500, Taiwan; 2 Institute of Biomedical Sciences, Academia Sinica, Taipei 115, Taiwan; 3 Bioinformatics Program, Taiwan International Graduate Program, Academia Sinica, Taipei 115, Taiwan; 4 Institute of Statistical Science, Academia Sinica, Taipei 115, Taiwan; 5 Graduate Institute of Clinical Medical Sciences, Chang-Gung University, Taoyuan 333, Taiwan

## Abstract

**Background:**

Association testing is a powerful tool for identifying disease susceptibility genes underlying complex diseases. Technological advances have yielded a dramatic increase in the density of available genetic markers, necessitating an increase in the number of association tests required for the analysis of disease susceptibility genes. As such, multiple-tests corrections have become a critical issue. However the conventional statistical corrections on locus-specific multiple tests usually result in lower power as the number of markers increases. Alternatively, we propose here the application of the longest significant run (*LSR*) method to estimate a region-specific p-value to provide an index for the most likely candidate region.

**Results:**

An advantage of the *LSR *method relative to procedures based on genotypic data is that only p-value data are needed and hence can be applied extensively to different study designs. In this study the proposed *LSR *method was compared with commonly used methods such as Bonferroni's method and FDR controlling method. We found that while all methods provide good control over false positive rate, *LSR *has much better power and false discovery rate. In the authentic analysis on psoriasis and asthma disease data, the *LSR *method successfully identified important candidate regions and replicated the results of previous association studies.

**Conclusion:**

The proposed *LSR *method provides an efficient exploratory tool for the analysis of sequences of dense genetic markers. Our results show that the *LSR *method has better power and lower false discovery rate comparing with the locus-specific multiple tests.

## Background

Recently whole genome association studies (WGA) with high density SNP data are becoming popular due to new technology in genotyping (e.g., Affymetrix and Illumina) [1-4]. Optimal study design in whole genome association remains unresolved although the two-stage association test design has gained popularity [5,6].

In order to improve power, many samples have been used in WGA studies; however the cost for such studies is still expensive even though the expense of genotyping has dropped significantly. One problem common of WGA studies is a high false positive rate if the direct method, based on the simple chi-square test for association, is performed without any correction for multiple testing. By far, the most commonly used remedy is the Bonferroni approach. However, its overly conservative correction might result in power reduction. There have been several methods proposed to circumvent the problem of the over correction of multiple testing procedures, e.g. false discovery rate (FDR) methods [7-9] that control the expected proportion of false rejections, or False Positive Report Probability (FPRP) [10] which requires explicit consideration of the prior probability for each hypothesis under association. Although the FPRP method aims to provide a higher power than traditional association test, the need for priors is not always possible. The FDR controlling method was originally designed for multiple comparisons of independent tests [7], and was then later extended to dependent cases under certain conditions, e.g. assuming the test statistics are either equally positively correlated and normally distributed, or having satisfied so-called the positive regression dependency [11]. As for the high density SNP data that were considered in this study, the aforementioned assumptions need to be justified in advance.

Most direct association tests are locus-specific and therefore seldom account for the association of different markers. Different markers usually have heterogeneous genetic backgrounds, such as allele frequency and marker characteristics. Single-point inference sometimes yields a misleading conclusion for an overall phenomenon. Methods considered multiple markers simultaneously and included logistic regression analysis, haplotype analysis [12,13], global significance method [14], multivariate association analysis [15], and the consideration of gene-gene interactions [16].

Instead of testing the significance of a single marker, our novel method tests the significance of all markers within a defined region, and therefore can be regarded as a simultaneous test for multiple markers that account for the dependence of close genetic markers.

The proposed "longest significant run" (*LSR*) method is a two-stage procedure. The first stage conducts conventional association tests, such as the chi-square test for the case-control design or the transmission disequilibrium test [17] for the family-based design. Based on the pre-specified size of a given test, the p-value of each test is converted into a zero/one indicator (1 for significance or 0 otherwise). In the second stage, this binary sequence is scanned for the longest region of consecutive 1s (hence "longest significant run") and the results determine whether or not the run is inordinately long or simply a random pattern.

This region-specific testing procedure is motivated by the dependence of association tests on dense markers: if a disease susceptibility gene lies in a specific region, then the disease gene and the nearby markers will show a relatively positive trend of association (i.e., linkage disequilibrium (LD)). A special non-random pattern (i.e., a cluster of positive signals) indicates that disease genes may be included in the candidate region. The evidence supporting such a non-random pattern is then evaluated with the magnitude of the longest run of consecutive significance.

There is a long list of applications for longest run statistics [18,19], one of which concerns the alignment and testing of the homology among DNA sequences. Considering two aligned sequences with length n, a match of locus between the sequences is assigned to be "1", and a mismatch is "0". More homologous sequences should have larger longest matching subsequences (run) than others [20], and its corresponding probability is used as an important reference to the homology between two sequences.

Given a similar concept, we use the longest significance run to find a region that is most likely to harbour a disease susceptibility gene in association studies. The dependent structure of the binary sequence is obtained from association tests by considering it as a discrete-time Markov chain model. Using extensive simulations, we demonstrate that the LSR approach provides a reasonable model of dependence for association test results whereby the false-positive and false-negative rates are all controlled effectively. The program in *R *code for *LSR *method is available (Additional File 1).

## Results

The program SIMLA version 3.1 (SIMulation of pedigree data for Linkage and Association studies)[21] was used to simulate family-structure genetic data. The program is available [22]. In the process, we generated 1000 trio families with genotypic data for SNP (single nucleotide polymorphism) markers. To determine the effects of the number of SNP markers, we considered two cases for 50 and 100 SNPs in a candidate region. To mimic the scenario of dependent markers, we considered a dense intermarker distance with a mean of 6 kb and a standard deviation of 10.7 kb. Note that in 500 K Affymetrix data, marker distance has an average of 5.8 kb and a standard deviation of 10.7 kb. In the simulation, the intermarker distance is generated from a left-truncated normal distribution with mean 6 kb and standard deviation 10.7 kb. Under the setup, the distribution of intermarker distance is similar to that of real Affymetrix data in the aspects of mean and standard deviation. Furthermore, the prevalence of the disease in the simulation was set as 0.1, and genetic relative risk was set to be 2.0, 2.5 and 3.0. The allele frequencies were 0.075 and 0.1, and an additive disease model was assumed. Note that the use of high relative risk is for observing contrasting powers between the methods under the stringent definition we used for detecting true positive markers (described in next sections). Under a looser definition, the relative risk can be lowered to 1.5 or less.

In the simulation, the location of disease gene was assigned to be within the two central markers, and the disease haplotype was comprised by 15 markers clustering around the disease gene. We had also tried randomly assigning the location of disease genes to be within any non-boundary markers, and found similar results. We considered three haplotype scenarios: (1) three common haplotypes each with a frequency of 30%, (2) two common haplotypes each with a frequency of 45%, and (3) two haplotypes with frequencies of 85% and 5%. Since the results of the three scenarios were similar, we present the scenario of three-common-haplotype.

During the first stage of the *LSR *method, the transmission disequilibrium test from the TDT procedure of SAS/GENETIC package release 8.2.39 [23] was used to test the locus-specific association for each marker. The output p-values {${\hat{p}}_{j}$, *j *= 1, Λ *n*} were transformed into a sequence of significance indicators {*X*_*j*_, *j *= 1,Λ,*n*} based on a test size *α*_1_, where *X*_*j *_is 1 if the test of the *j*^th ^marker is significant and 0 otherwise. In the second stage, we scanned this binary sequence to identify the longest perfect run of 1, *L*_0_, the nearly perfect run of 1 allowing one zero within, namely the *L*_1 _and the run of 1 allowing two zeros within, namely the *L*_2_. For each of these three *LSR *statistics, the corresponding tail probabilities were calculated using the method of Chang *et al*. [20].

### Testing statistics

Although allowing more interruptions (larger k) seems to make the *LSR *approach more plausible, it is helpful only if it can bridge up nearby but separated clusters of "1's". On the other hand, containing irrelevant markers may complicate further analysis to identify the target gene. To compromise the situations we defined the testing statistics, *LSR*_*{k}*_, as *L*_*i *_with minimal tail probability, for i from 1 to k and considered only kϕ2. In the simulation, we compared our method with the Bonferroni method, the popular correction used for multiple tests, by evaluating three quantities, namely the false-positive rate, power, and false discovery rate in the following three sections.

### False-positive rate

According to our setup in the simulation, markers in a sequence can be divided into three categories: 2 target markers where the disease gene lain between them, 13 nearby markers in linkage disequilibrium (LD) with the 2 target markers, and other markers that are in linkage equilibrium (LE). For the purpose of stringency, a true positive detection was considered only if at least one of the two target markers was significant under the criterion of each method. For Bonferroni's method, the criterion is that a target marker's p-value < α_2_/n. For the FDR controlling method, Benjamini and Hochberg [7] proposed the following criterion. The *n *single marker p-values were sorted from smallest to largest: *P*_(1) _⋯ *P*_*(n)*_. Starting from *P*_(*n*)_, we compared *P*_(*i*) _with i·α_2_/n. This process was continued as long as *P*_(*i*) _> i·α_2_/n. If k is the turn around point, then significance is declared if a target marker corresponds to the k^th ^smallest p-value. For our *LSR *method, the criterion was that a target marker is covered by a significant *LSR *(p-value< α_2_). On the other hand, a false positive case was considered if a LE marker was detected under each of the three criteria described above. If a non-target LD marker was selected, neither a false positive nor a true positive would be counted.

The false-positive rate is the probability that a test mistakenly rejects the null hypothesis. In our case, it is the rate at which a test falsely detects disease susceptibility genes where none exists. In our first part of the simulation, no disease gene was assumed. Table 1 lists the corresponding false-positive rates for the two *LSR *approaches and Bonferroni approach based on 200 replications for each of the eight scenarios (2 disease gene allele frequencies, 2 relative risk, 2 marker numbers). The test size in the first stage was *α*_1 _= 0.1, and the test size was *α*_2 _= 0.05 in the second stage. We found that all the false-positive rates were under or similar to the pre-specified nominal test size *α*_2_, indicating that all the methods adequately controlled the false-positive rate.

**Table 1:** Comparisons of false positive rate while the SNP data are in linkage disequilibrium.

Marker number	Relative risk	Allele frequency	False positive rate		
			
			Bonferroni	LSR_1_	LSR_2_
50	2.0	0.075	0.035	0.01	0.015
	2.0	0.1	0.04	0.01	0.01
	2.5	0.075	0.05	0.025	0.025
	2.5	0.1	0.04	0.015	0.015

100	2.0	0.075	0.04	0.015	0.015
	2.0	0.1	0.025	0.035	0.055
	2.5	0.075	0.045	0.025	0.035
	2.5	0.1	0.02	0.025	0.035

Simulation was based on 200 replications with sample size 1000, under additive disease model. Screening criteria for LSR:α_1 _= 0.10, and level of significance for all methods:α_2 _= 0.05

### Power and false discovery rate

Statistical power is the probability that a test correctly rejects the null hypothesis. In our case, it is the probability that a method correctly accesses at least one of 2 target markers with statistical significance. On the other hand, the false discovery rate is the fraction of false positives among all tests declared significant.

**Figure 1:**
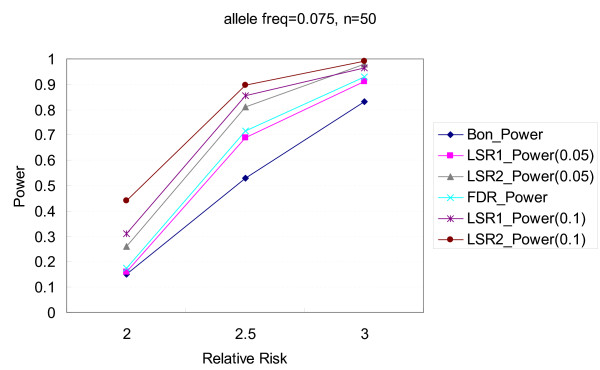
**Comparisons of power among the procedures of Bonferroni correction, two LSR methods, and FDR control method**. The allele frequency is 0.075, and the number of markers is 50. Two criteria, α_1 _= 0.05 and 0.1, are used for the LSR method.

**Figure 2:**
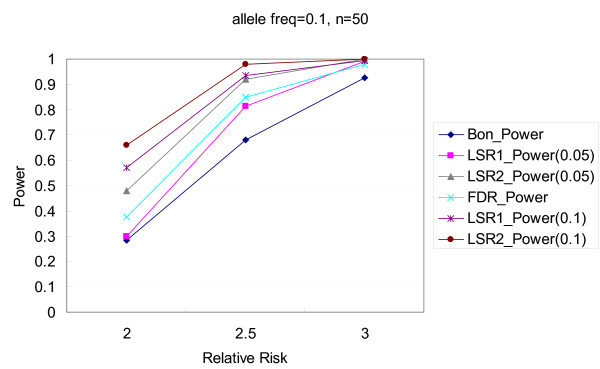
**Comparisons of power among the procedures of Bonferroni correction, two LSR methods, and FDR control method**. The allele frequency is 0.1, and the number of markers is 50. Two criteria, α_1 _= 0.05 and 0.1, are used for the LSR method.

**Figure 3:**
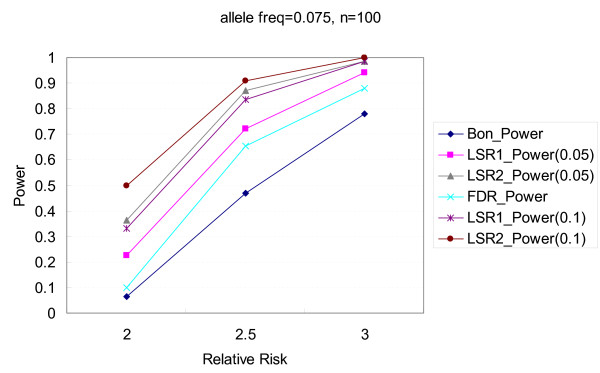
**Comparisons of power among the procedures of Bonferroni correction, two LSR methods, and FDR control method**. The allele frequency is 0.075, and the number of markers is 100. Two criteria, α_1 _= 0.05 and 0.1, are used for the LSR method.

**Figure 4:**
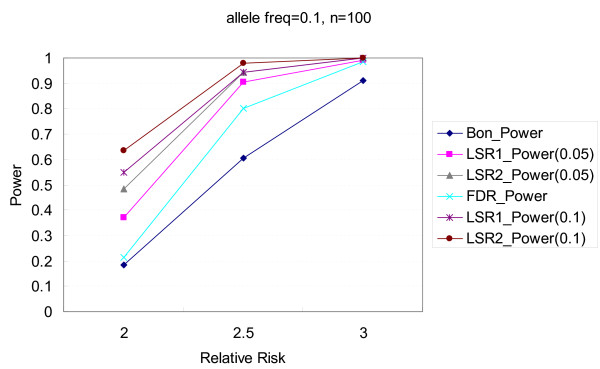
**Comparisons of power among the procedures of Bonferroni correction, two LSR methods, and FDR control method**. The allele frequency is 0.1, and the number of markers is 100. Two criteria, α_1 _= 0.05 and 0.1, are used for the LSR method.

Figures 1, 2, 3, 4 illustrate the performance of Bonferroni and *LSR *methods given different nominal test sizes. The test size in the first stage for *LSR *was *α*_1 _= 0.05 and 0.1 and the test size in the second stage was *α*_2 _= 0.05. Higher level of significance (0.1) at screening stage resulted in better power than that of *α*_1 _= 0.05, in trade of a slightly higher false discovery rate. *LSR*_2 _had better performance in both power and false discovery rate than *LSR*_1_. The stringent definition of a "run" for *LSR*_1 _yields a relatively narrow candidate region that was more likely to miss the target markers. Both *LSR*_1 _and *LSR*_2 _had better power and false discovery rate than those from the Bonferroni approach among all the scenarios that we tried. *LSR*_1 _has similar power to that of the FDR controlling method, but better false discover rate. In summary, in terms of power, *LSR*_2 _> *LSR*_1_~ FDR controlling method > Bonferroni; and in terms of false discovery rate, *LSR*_2_~*LSR*_1 _~<FDR controlling method < Bonferroni. The detailed results are available in Tables 2 and 3.

**Table 2:** Comparisons of power and false discovery rate among the procedures of Bonferroni correction and two LSR methods.

Marker number	Relative risk	Allele frequency	Power			False discovery rate			Length*	
					
			Bonferroni	LSR_1_	LSR_2_	Bonferroni	LSR_1_	LSR_2_	LSR_1_	LSR_2_
50	2.0	0.075	0.15	0.16	0.255	0.14	0	0	4.27	5.72
	2.0	0.1	0.285	0.3	0.48	0.07	0	0	5.765	7.645
	2.5	0.075	0.53	0.69	0.81	0.06	0	0	8.7	10.39
	2.5	0.1	0.68	0.815	0.92	0.03	0.005	0.005	9.695	11.74
	3.0	0.075	0.83	0.91	0.98	0.04	0	0	10.7	12.9
	3.0	0.1	0.925	0.99	1	0.03	0	0	11.5	14.0

100	2.0	0.075	0.065	0.225	0.365	0.28	0.015	0.02	4.34	5.715
	2.0	0.1	0.185	0.37	0.485	0.18	0.005	0.02	5.685	7.1
	2.5	0.075	0.47	0.72	0.87	0.11	0.005	0.025	8.435	10.26
	2.5	0.1	0.605	0.905	0.945	0.05	0.01	0.005	10.07	11.33
	3.0	0.075	0.78	0.94	0.985	0.02	0.005	0.005	10.9	13.0
	3.0	0.1	0.91	0.99	1	0.03	0.005	0.005	11.6	13.8

Simulation was based on 200 replications with sample size 1000, and SNP data were generated under additive disease model with 15 markers in linkage disequilibrium. Screening criteria for LSR:α_1 _= 0.05, and level of significance for all methods:α_2 _= 0.05

**Table 3:** Comparison of power and false discovery rate among the procedures of Bonferroni correction and two LSR methods.

Marker number	Relative risk	Allele frequency	Power			False discovery rate			Length*	
					
			Bonferroni	LSR_1_	LSR_2_	Bonferroni	LSR_1_	LSR_2_	LSR_1_	LSR_2_
50	2.0	0.075	0.15	0.31	0.44	0.14	0	0.005	5.95	7.765
	2.0	0.1	0.285	0.57	0.66	0.07	0	0	7.74	9.66
	2.5	0.075	0.53	0.855	0.895	0.06	0.005	0.005	10.22	12.05
	2.5	0.1	0.68	0.935	0.98	0.03	0	0	11.23	13.215
	3.0	0.075	0.83	0.965	0.99	0.04	0.005	0	11.9	13.9
	3.0	0.1	0.93	0.995	1	0.03	0	0	12.6	14.6

100	2.0	0.075	0.065	0.33	0.5	0.28	0.03	0.04	5.91	7.51
	2.0	0.1	0.185	0.55	0.635	0.18	0.02	0.055	7.28	9.055
	2.5	0.075	0.47	0.835	0.91	0.11	0.015	0.04	9.82	11.755
	2.5	0.1	0.605	0.945	0.98	0.05	0.005	0.005	11.33	13.475
	3.0	0.075	0.78	0.985	1	0.02	0.005	0	12.2	14.1
	3.0	0.1	0.91	1	1	0.03	0	0	12.8	14.6

Simulation was based on 200 replications with sample size 1000, and SNP data were generated under additive disease model with 15 markers in linkage disequilibrium. Screening criteria for LSR:α_1 _= 0.10, and level of significance for all methods:α_2 _= 0.05

The good performance of LSR does not indicate that it can completely replace the marker-specific test procedures like the Bonferroni approach. Rather, we consider LSR as a useful screening tool to find a smaller region that possibly contains the disease susceptibility gene. After the region is identified, each maker within the region still needs to be examined biologically. At this stage, *LSR*_2_, although better than *LSR*_1 _with respect to both power and false discovery rate, pays a token for retaining more makers for further examining. Analogue can be inferred in comparing the performance of using *α*_1 _= 0.1 over *α*_1 _= 0.05 as the threshold in the first stage of LSR, where the former usually includes more markers.

The tail probabilities of *LSR *were calculated under the null hypothesis of no disease marker. In the simulation, Markov independence was assumed for the binary sequences under null hypothesis. Nonetheless, the method can also be applied to the scenario of first order Markov-dependency, if the corresponding transition matrix can be assumed or estimated. We provide a robust approach to estimate the dependency structure of the sequence using the concept of sliding window, which is described in the method section. The method was applied on the following authentic data as illustration.

### Demonstrating the applications of our method to two authentic data sets

#### Example of psoriasis data

We assessed the practical application of the *LSR *method using an authentic genetic data set collected for a psoriasis study [24]. Psoriasis is a common chronic skin disorder characterized by inflammation and scaling. Recent studies indicate a significant association of important psoriasis predisposing loci with chromosome 17 [24,25]. Helms *et al*. [24] collected 242 European nuclear families comprising 572 psoriasis cases and genotyped 123 genetic markers on chromosome 17q25. The family-based association test TDT-AE [26] was applied to locate the psoriasis susceptibility genes.

In the first stage of *LSR *procedure, we used *α*_1 _= 0.05 as a test size to convert the sequence of p-values into a binary sequence. Under null hypothesis assuming Markov independency, the LSR obtained a significant p-value <0.001, and the region of 12 consecutive markers identified by *L*_0 _captured two functional genes *SLC9A3R1 *and *DKFZPP564C103*, which are known to be psoriasis-related. The results are consistent with the finding of Helms *et al*. [24].

However, when we proceeded with the sequential goodness-of-fit tests proposed by Anderson and Goodman [27], the hypothetical assumption of {*X*_*j*_} being an independent sequence was rejected thereby supporting the hypothesis of it being a first order Markov dependence. Therefore, a sliding windows approach was applied with size of 40 markers and sliding distance of 10 markers to estimates of the transition matrix under the hypothesis of Markov dependency. The same region was identified by *LSR *with p-value 0.007 under the dependency assumption of null hypothesis. Similar results were produced when minor variations in window size and sliding distance were used.

In this example, *L*_1 _and *L*_2 _did not further extend the region of *L*_0_, therefore, *L*_0 _is sufficient to confirm this candidate region. On the other hand, in order to attain approximately evenly spaced intermarker distance for simple-count approach and avoid combining two distinct chromosome regions, we tried only using the results from the 5^th ^to the 86^th ^loci among the 123 markers. Moreover, we also excluded results from marker loci with less than 50 trios in the analysis to preclude possibly unreliable association results. It resulted in 78 loci included in the final analysis. The consequent analysis also came out with the same significant region with a p-value <0.001. Of particular note, all of the 123 markers had p-value >0.0001, therefore, none of them was significant after direct Bonferroni adjustment.

#### Example of asthma data

Asthma is a common chronic disease characterized by airway inflammation resulting in some symptoms such as the difficulty of breathing, attacks of wheezing and coughing, etc,. In the positional-cloning study of Allen *et al*. [28], there were 224 families containing 239 asthma children and 79 markers covering a region of 384 kb on chromosome 2q14. Immunoglobulin E often causes asthmatic inflammation and has been recognized to be an important concomitant factor of children asthma. Allen *et al*. [28] conducted the transmission disequilibrium tests to assess the relationship between marker loci with asthma and immunoglobulin E.

Using test size *α*_1 _= 0.05, the sequence of p-values was converted into a sequence of twenty-three 1s and fifty-six 0s:

**X **= [0000000011111110111110010011000100000101101011001000000000000000000000000000000].

The goodness-of-fit test suggested that the sequence follows a first order Markov chain. In the second stage of *LSR *method, we estimated the transition probabilities *η*_00 _and *η*_11 _to be 0.647 and 0.7, respectively, and identified *L*_1 _(from marker 543WTC21P at 191388 to 543WTC91P at 252279, details are listed in Additional File 2) with length 13 and yielded the corresponding p-value 0.0002. Note that *L*_1 _contains a significant candidate region of 60891 bp closed to an asthma-suspected gene *DPP10 *with functions of catalytic activity and dipetidyl-peptidase IV activity. The conclusion is consistent with Allen *et al*. [28] who found the highly significant STRP marker D2S308 at 261056 in the vicinity of our identified region.

## Discussion

As discussed by Rosenthal [29] and Zaykin *et al*. [30], a series of border-line significant results may together suggest significance. Therefore, it is likely that two consecutive p-values of 0.06 may suggest evidence against the null than one isolated p-value of 0.05. This phenomenon is often observed in the identification of candidate regions of complex disorders if the marginal effect of a disease allele is modest or minor with a few adjacent loci that are in LD. In this study, we propose the longest significant run, *LSR*, to estimate region-specific p-values while searching for disease susceptibility genes in gene mapping studies. The method transforms the p-values from locus-specific association tests (e.g., the transmission disequilibrium test or any other association test) to a binary sequence with the value '1' representing significance and '0' otherwise, and determines the *LSR *to identify the location of the target disease susceptibility gene. The statistical significance of *LSR *method can be accessed by imbedding the sequence onto a Markov chain. A sequential goodness-of- fit-test [27] can be used to justify the assumption that a sequence of indicator of p-values is Markov chain, simulations assuming no disease gene were carried out, and the test did not reject the null hypothesis of Markov independence 99.8% of the time (998 out of 1000). On the other hand, in the simulations assuming a disease gene with allele frequency of 0.075 and RR = 2.5 imbedded in a region flanking with dense markers, the test rejected the null hypothesis of Markov independence 92.4% of the time (924 Out of 1000) but did not reject the null hypothesis of first order of Markov chain 74.4% of the time (744 out of 1000). Likewise, the test did not reject the null hypothesis of first order Markov chain 82.2% of the time (822 out of 1000), assuming a disease susceptibility gene with allele frequency of 0.1. The allele frequencies in our simulations were assumed for the disease-causing alleles. The allele frequency for most markers in genome-wide association study is higher than 0.05, therefore we chose to use disease-causing allele frequencies to be 0.075 and 0.1. Other frequencies were also tested and found that the power was positively correlated with the allele frequency. This disease related marker was removed in all our subsequent analyses. Only results from additive disease model were reported since it was more plausible for complex diseases; we also examined dominant model which showed higher power and recessive model which showed lower power.

The *LSR *method avoids the controversial multiple-test adjustment required for locus-specific association tests, and thus has potential applications in exploratory data analysis. For example, if there were a few significant markers in a genome-wide association found by using single locus test [31], our proposed methods can be applied to screen out the most likely region for further biological examination. However, for an isolated significant marker, it is likely that the *LSR *method will miss the signal. Therefore, single-maker method and *LSR *method should be considered complementary.

In addition to the perfect *LSR*, we also propose a "nearly perfect" *LSR *in which a few interruptions (insignificance) are allowed within the run. Our simulation results suggest that the *LSR*s which allow for one or two interruptions within the run are generally more flexible and have some gain of testing power. The approach can be easily extended to allow more interruptions by modifying the imbedded transition matrix in Equation (5) in the method section.

An advantage of our proposed method relative to testing procedures based on genotypic data is that only p-value data are needed. This method can be adapted extensively to different study designs and testing procedures if reliable p-values are provided. An important application is meta-analysis which combines p-values from different studies. Due to the high sensitivity of *LSR *method, many published results with summarized p-values originally reported as insignificant can be reanalyzed by this method for more convincing conclusions.

**Figure 5:**
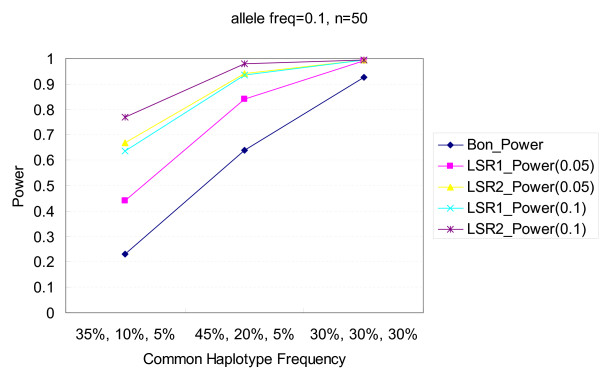
**Comparisons of power among the procedures of Bonferroni correction and two LSR methods under different common haplotype frequency scenarios**. The allele frequency is 0.1, and the number of markers is 50. Two criteria, α_1 _= 0.05 and 0.1, are used for the LSR method.

To explore the genetic structure of human genome, we downloaded the first 3,000 markers of chromosomes 1, 5, 10, 15 and 20, respectively, from the HAPMAP website [32]. According to Gabriel's criterion (95% CI of D' = 0.7, 0.98) [33], there are 672 LD blocks among the 15,000 markers. The mean frequency and standard deviation of the top haplotype are 0.55 and 0.19. They are 0.24 and 0.09 for the second haplotype and 0.12 and 0.07 for the third one. If the top two or three haplotypes are defined as the "common haplotypes", about 62% of the 672 LD blocks have a cumulative frequency of 90% and about 99.4% of the blocks have a cumulative frequency of 50%. To compare the powers of our proposed method between optimal and modest LD scenarios, we used two settings of haplotype frequencies, (85%, 5%) and (30%, 30%, 30%) for the former and a setting of (35%, 10%, 5%), where the frequencies were roughly those mean frequencies minus one respective standard deviation, for the latter. As expected, the former scenario with optimal LD block resulted in better power for all methods in our simulation than the latter with modest LD block. The power of *LSR*_2 _for this modest LD scenario is about 77% assuming 0.1 disease allele frequency, RR = 3 and α_1 _= 0.1. The power is about 23% for the Bonferroni procedure. The results for the different LD scenarios comprised of 3 common haplotypes are also presented in Figure 5.

We further compared the power of our proposed methods with those of haplotype analyses carried out by the FBAT program [34] assuming RR = 2 and disease allele frequency of 0.075. For the optimal LD scenarios, the multiple-degree of freedom (m-df) and single-degree of freedom (1-df) haplotype tests yield powers of 0.46 and 0.232, respectively, for the setting of (85% and 5%). The powers for m-df and 1-df are 0.35 and 0.176, respectively, for the setting of (30%, 30%, 30%). For the modest LD scenario of (35%, 10%, 5%), the powers for m-df and 1-df are 0.18 and 0.126, respectively. The power of LSR_2 _is 0.44 with α_1 _= 0.1 and the power of Bonferroni procedure is 0.15. Therefore, the power of LSR_2 _is about the same as the power of haplotype analyses under optimal LD scenarios but outperforms under modest LD scenario. Powers using other values of RR were also calculated. In general, powers of both haplotype analyses and LSR increase assuming higher values of RR.

Our simulation study demonstrates that all the methods adequately control the false-positive rate, however, the *LSR *methods, in particular, *LSR*_2_, had better performance in both power and false discovery rate than those for the Bonferroni and FDR controlling methods. Moreover, as the number of markers increases, the power of both the Bonferroni and FDR controlling method drops significantly, whereas the power of *LSR *remains high and may even increase slightly.

Further studies for potential extensions of the LSR method are currently under development. Firstly, the *LSR *method transforms continuous p-values to a dichotomous random sequence of 0s and 1s, which may cause a loss of information. This limitation implies that some isolated disease susceptibility loci cannot be identified by the *LSR *method. A model based on a more complex continuous-state stochastic process constitutes an alternative that would exploit more information. Secondly, in our simulation, we assumed that there is only one disease susceptibility gene in the region of interest. However, the *LSR *method will miss some isolated target genes when a region contains two or more. One remedy is to extend the *LSR *theory to incorporate the second or third longest significant run.

The remarkable advances in genotyping technology have facilitated the pursuit of genome-wide association mapping. The increased density of available SNP markers provides finer resolution and higher statistical power for gene mapping. For example, the average distance between two SNPs using the 100 K assay of Affymetrix [35] is 24 kb. The intermarker distance is only 5.8 kb for the 500 K chips. We foresee that the multiple tests correction will remain a crucial issue as the number of genetic markers becomes very large. Our method can be applied to such data in combination with the sliding window method to screen out candidate regions associated with the disease genes.

## Conclusion

In summary, the *LSR *method provides an efficient exploratory tool for the analysis of sequences of dense genetic markers thereby complementing current locus-specific methods. Our simulation study demonstrates that the *LSR *method has reasonable statistical power and avoids the over-correction problem that plagues most of the locus-specific methods. When applied to actual genetic data, the *LSR *method successfully confirmed the location of two important psoriasis-associated genes and an asthma-related gene. The application to genome-wide screening studies may further enhance the proposed *LSR *method.

## Methods

Suppose that there are a total of *n *genetic markers in a study. The *LSR *method contains two main stages, the first of which transforms the results from *n *locus-specific association tests into a sequence of significance indicators and the sequence is used in the second stage to locate the *LSR *and compute its tail probability.

### The first-stage procedure

Association tests are applied to examine the significance of associations between genetic markers and a disease susceptibility gene, and the corresponding p-values (significance probabilities) of the tests are denoted by {${\hat{p}}_{j}$, *j *= 1, Λ *n*}. Under the pre-specified test size, *α*_1_, we consider an indicator function of the test on marker *j*: *X*_*j *_= *I*[${\hat{p}}_{j}$, <*α*_1_] where *I *[*A*] = 1 if event *A *holds, and *I *[*A*] = 0 otherwise.

In the following hypothetical example of 15 genotyped genetic markers (*n *= 15), the corresponding p-value vector of 15 association tests is $\hat{P}=[{\hat{p}}_{1},\cdots ,{\hat{p}}_{15}]$ = [0.30, 0.04, 0.03, 0.04, 0.12, 0.04, 0.02, 0.01, 0.35, 0.50, 0.02, 0.03, 0.04, 0.45, 0.04] Based on the setting of *α*_1 _= 0.05, we obtain

(1) **X **= [*X*_1_, L, *X*_15_] = [0, 1, 1, 1, 0, 1, 1, 1, 0, 0, 1, 1, 1, 0, 1],

where markers 2, 3, 4, 6, 7, 8, 11, 12, 13, and 15 show significant association with the disease susceptibility gene.

### The second-stage procedure

Before applying the LSR method, the assumption of Markov chain for the sequence of the indicator of p-values can be checked by carrying out the goodness-of-fit test of Markov Chain [27]. Assuming that the binary random sequence {*X*_*j*_, *j *= 1,Λ,*n *} to be a first order Markov chain stochastic process satisfying

(2) Pr{*X*_*j *_= *s*_*j *_| *X*_*j*-1 _= *s*_*j*-1_,Λ, *X*_1 _= *s*_1 _= Pr{*X*_*j *_= *s*_*j *_| *X*_*j*-1 _= *s*_*j*-1_},

where *s*_*k *_∈ {0,1} = insignificance, significance}, *k *= 1,Λ, *n *. Let the initial probability be Pr{*X*_1 _= 1} = *η*_1 _and Pr{*X*_1 _= 0} = 1 - *η*_1_, then the transition probability matrix is

(3) $$P_{j}=\left[\begin{array}{cc} \mathrm{Pr}\{X_{j}=0|X_{j-1}=0\} & \mathrm{Pr}\{X_{j}=1|X_{j-1}=0\}\\ \mathrm{Pr}\{X_{j}=0|X_{j-1}=1\} & \mathrm{Pr}\{X_{j}=1|X_{j-1}=1\} \end{array}\right]=\left[\begin{array}{cc} \eta_{00j} & \eta_{01j}\\ \eta_{10j} & \eta_{11j} \end{array}\right],$$

where *η*_00*j *_+ *η*_01*j *_= 1 and *η*_10*j *_+ *η*_11*j *_= 1, *j *= 2,Λ,*n*. This model assumes conditional independence and is well established in a variety of fields.

An inordinately long *LSR *indicates possible associations between markers and a disease susceptibility gene. Due to random error, low heterozygosity or other unknown reasons, not all markers linked to the disease gene show positive association. Hence, it is sensible to allow for a few interrupting 0s (insignificances) in a run. We denote the more flexible run as a "nearly perfect run" in contrast to a perfect run that does not contain 0s. A unifying notation of the length of an *LSR *is *L*_*k*_, where the subscript *k *denotes the number of interrupting 0s. When *k *= 0, the case reduces to a perfect run. In the example in Equation (1), *L*_0 _= 3, *L*_1 _= 7, *L*_2 _= 8 and *L*_3 _= 12.

#### Consider the following hypotheses

H_0_: "there is no disease susceptibility gene in the sequence of markers", versus

H_1_:"there is at least one disease susceptibility gene in the sequence".

According to the argument above, an inordinately long *LSR *(i.e. large *L*_*k*_) indicates a non-random cluster of significant associations with nearby markers, suggesting that disease susceptibility genes may exist in the candidate region. For a pre-chosen *k*, an intuitive rule to reject H_0 _is *L*_*k *_> m*, where the constant m* is the minimum integer of *m *satisfying the following inequality

(4) Pr{*L*_*k *_≥ *m *| H_0_} ≤ *α*_2_

for a test size *α*_2_. To determine the critical region, we need to calculate the tail probability of testing statistic *L*_*k*_.

### Distributions of LSR statistics

There has been a long history of studies on the distribution of the length of the longest run. Erdõs and Révész [36] considered a binary sequence from a coin-tossing game, with outcomes of head (coded as "1") and tail ("0"). The p-value of *L*_0 _of *LSR *is analogous to the tail probability of their longest head-run. Fu and Koutras [19] have provided an algorithm to calculate the exact probability of *L*_0_. Arratial *et al*. [37] and Karlin *et al*. [38] have provided asymptotic results on the distribution *L*_*k*_, however, the former method [37] is valid only for independent sequences, whereas the latter [38], although allowing dependency, yields a large bias for the estimation of tail probability when *n *is of moderate size (e.g., *n *= 200) [20].

Chang *et al*. [20] provided an algorithm and software to calculate the exact probability of *L*_*k *_by the following formula:

(5) $$\mathrm{Pr}\{L_{k}\geq m\}=1-u\times (\prod_{t=1}^{n}\Lambda_{t})\times v^{T},$$

where u = [1,0,Λ,0], *ν *= [1,Λ,1,0] and the exact form of the imbedded transition matrix Λ_*i *_is a function of *η*_1 _and *η*_*sti *_s in Equation (3). By considering a homogeneous Markov chain, the subscript *i *can be suppressed and Λ_*i *_and *η*_*sti *_can be reduced to Λ and *η*_*st*_. We estimated the initial probability by the proportion of the occurrence of "1" in {*X*_*j*_, *j *= 1,Λ,*n*}, i.e., ${\hat{\eta }}_{1}$ = (number of "1")/n. Under null hypothesis *H*_0 _of independency, {*X*_*j *_is assumed to be of no special pattern and the transition probabilities *η*_1*t *_and *η*_0*t *_can be replaced by ${\hat{\eta }}_{1}$ and 1 - ${\hat{\eta }}_{1}$. For dependent sequences, we propose the following approach.

### Estimation of dependency structure under null hypothesis

To examine the Markov chain assumption on sequence {*X*_1_...*X*_n_}, we used the following sequential goodness-of-fit tests proposed by Anderson and Goodman [27]:

(i) H_0_: independent sequence vs. H_1_: first order Markov chain

(ii) H_0_: first order Markov chain vs. H_1_: second order Markov chain

If the first order Markov chain hypothesis is accepted, we suggest using the following approach to estimate the dependency structure and calculate the p-value.

Consider a set of sliding windows of fixed size *w *and sliding distance *d*, *d *<*w *<*n*, under proper choices of *d *and *w*, the windows {*X*_1_...*X*_w_}, {*X*_1+*d*_...*X*_w+d_}, ..., {*X*_1+sd_...*X*_w+sd_} will cover nearly the whole sequence, where *s *is the largest integer smaller than or equal to (*n-w*)/*d*. For the subsequence in the *i-*th window, the transition probabilities *η*_*st *_(*i*) = *P*(*X*_*i*+1 _= *t *| *X*_*i *_= *s*), where *s*, *t *= 0,1, are estimated by its maximal likelihood estimator, namely, ${\hat{\eta }}_{11}$(*i*) = (number of consecutive (1,1) pairs)/(number of 1s), and ${\hat{\eta }}_{00}$ (number of consecutive (0,0) pairs)/(number of 0s), for *i *= 1 to *d*+1. For a homogeneous first-order Markov chain, we estimate ${\hat{\eta }}_{11}$ and ${\hat{\eta }}_{00}$ by the medians of ${\hat{\eta }}_{00}$(*i*)'s and ${\hat{\eta }}_{11}$(*i*)'s, respectively, and ${\hat{\eta }}_{10}=1-{\hat{\eta }}_{11}$ and ${\hat{\eta }}_{01}=1-{\hat{\eta }}_{00}$.

The validity of the sliding window approach is based on the assumption that most of the widows contain no disease markers, and therefore the sequences within the set of sliding windows of fixed size *w *can be used to estimate the transition probabilities. After ${\hat{\eta }}_{st}$(*i*)'s are estimated from each window, the robustness of median can diminish the influence from those few windows which might contain disease markers. With the estimated *η*_*st*_'s, tail probabilities of *LSR *statistics can be calculated using (5), and then the null hypothesis of no disease gene is rejected if *L*_*k *_≥ *m*_*α*_, where *m*_*α *_is a threshold such that Pr{*L*_*k *_≥ *m *_*α*_|*H*_0_} = *α*.

To summarize, we performed simulation studies to investigate the false-positive rate, the power, and the false discovery rate of the proposed *LSR *method, and we compared the results with those from Bonferroni method.

## Authors' contributions

I–BL developed method and directed simulations. Y–HL and Y–CL conducted simulations and data analyses. H–CY drafted the manuscript. C–JC proposed the original idea and directed simulations. CSJF directed simulations, drafted manuscript and supervised the project. All authors have read and approved the final manuscript.

## Supplementary Material

Additional file 1 The program in *R *code for *LSR *method. The LSR program in R code. Click here for file

Additional file 2 The details of the markers in the 0–1 sequence. The marker position and marker name of the 0–1 sequence. Click here for file
