# Supplementary material for: Using the longest significance run to estimate region-specific p-values in genetic association mapping studies
Source: BMC Bioinformatics. 2008 May 27;9:246. doi: 10.1186/1471-2105-9-246 (PMC2430975; doi:10.1186/1471-2105-9-246)
Supplement: Additional file 1 — The program in R code for LSR method. The LSR program in R code. [file 1471-2105-9-246-S1.doc]

### Additional file 1 –The program in *R* code for *LSR* method

#Main program: LSR.indep(pv, threshold, k)

#Calling the following subroutines:

#Lk.length(a, k)

#Lk0to3(ps, pss, pff, n, d, k)

#Lk.waterman(a,k)

#Lk.0<-function(ps, pss, pff, n, d)

#Lk.1<-function(ps, pss, pff, n, d)

#Lk.2<-function(ps, pss, pff, n, d)

#Lk.3<-function(ps, pss, pff, n, d)

####################### Main program ###########################

LSR.indep<-function(pv, threshold, k)

{

# this program reports the tail probability of Lk (longest k-interrupted run)

# for k=0-3 of a binary sequence obtained from a pvalue seq cut by threshold#

# output: Lk, its exact and asymptotic p-value under independent assumption#

# the main program need the following subroutine to work :

# longfexact, Lk.0-Lk.3

kk<-(0:k)

dd <- rep(0,length(kk))

pss <- rep(0,length(kk))

pff <- rep(0,length(kk))

a<-c()

for(i in 1:length(kk)){

n <- length(a)

if(sum(a==1)==0){dd[i]<-0

coord<-0}

else if(sum(a==1)!=0){

temp <- Lk.length(a, kk[i])

dd[i] <- temp$d

coord <- temp$coord

}

d1 <- dd[i] + 1

ps <- sum(a)/n

pss[i]<-ps

pff[i]<-1-ps

}

if (k==0)

{

if(sum(a==1)==0){

d<-0

coord<-0}

else if(sum(a==1)!=0){ n <- length(a)

temp <- Lk.length(a, k)

d <- temp$d

coord <- temp$coord }

kpb <- Lk0to3(pss[k+1], pss[k+1], pff[k+1], n, dd[k+1],k)}

if (k==1)

{

if(sum(a==1)==0){

d<-0

coord<-0}

else if(sum(a==1)!=0){ n <- length(a)

temp <- Lk.length(a, k)

d <- temp$d

coord <- temp$coord }

kpb <- min(Lk0to3(pss[k], pss[k], pff[k], n, dd[k],0), Lk0to3(pss[k+1], pss[k+1], pff[k+1], n, dd[k+1],1))}

if(k==2){

if(sum(a==1)==0){d<-0

coord<-0}

else if(sum(a==1)!=0){ n <- length(a)

temp <- Lk.length(a, k)

d <- temp$d

coord <- temp$coord }

ps.1<-round((ps-0.0049999)/2,dig=2)*2

sit.d <- which(Lk.length(a,2)$d==prob.m[,1])

sit.p1 <- which(ps.1==prob.m[1,])

temp2 <-prob.m[sit.d,sit.p1]

kpb <- min(Lk0to3(pss[k-1], pss[k-1], pff[k-1], n, dd[k-1],0), Lk0to3(pss[k], pss[k], pff[k], n, dd[k],1), temp2)

}

apb <- Lk.waterman(a,k)

bonfe1 <- min(length(pv)*min(pv[25],pv[26]), 1)

#bonfe2 <- min(length(pv)*min(pv[21:30]), 1)

cat("length of Lk:",d, "\n")

cat("range of Lk:", coord,"\n")

cat("for Markov independent model,", "\n")

cat("exact p-value P(Lk>=d)=", round(kpb, dig=5), "\n")

cat("asymptotic p-value P(Lk>=d)=", round(apb,dig=5), "\n")

cat("Bonferroni adjusted p-value=", round(bonfe1,dig=5), "\n")

#cat("Bonferroni adjusted p-value=", round(bonfe2,dig=5), "\n")

outputLk<-round(c(kpb, apb, bonfe1, d, coord), dig=5)

}

######################### subroutine 1 ###############################

Lk0to3<-function(ps, pss, pff, n, d, k)

{

if(k == 0)

Lk.0(ps, pss, pff, n, d)

else if(k == 1)

Lk.1(ps, pss, pff, n, d)

else if(k == 2)

Lk.2(ps, pss, pff, n, d)

else if(k == 3)

Lk.3(ps, pss, pff, n, d)

else if(k > 3)

cat("This program allows 3 mismatches at most", "\n")

}

######################### subroutine 2 ###############################

Lk.length<-function(a, k)

{

#this program reports the max length of well-matching (1) run, allowing

#k mismatches with in a binary sequence a.#

# output: Lk

if(k > 5) cat("k has to be less than 6", "\n")

n <- length(a)

j <- 0

x <- NULL

y <- NULL

for(i in 1:(n - 1)) {

if(a[i] != a[i + 1]) {

j <- j + 1

x[j] <- i

}

}

L1 <- matrix(0, 1, j)

L2 <- matrix(0, 1, j)

L3 <- matrix(0, 1, j)

L4 <- matrix(0, 1, j)

L5 <- matrix(0, 1, j)

L <- matrix(1, 2, j)

if(j == 0) {

if(a[1] == 1) {

y[1] <- n

L[1] <- n

}

if(a[1] == 0) {

y[1] <- n

L[1] <- 0

}

}

if(j > 0) {

y[1] <- x[1]

y[j + 1] <- n - x[j]

if(j > 1) {

for(j in 2:j) {

y[j] <- x[j] - x[j - 1]

}

}

t <- 1

if(a[1] == 1) {

if((2 * t + 1) > (j + 1)) {

L <- y[1]

}

while((2 * t + 1) <= (j + 1)) {

s1 <- y[2 * t - 1] + y[2 * t + 1]

s2 <- y[2 * t]

L1[t] <- (s1 + s2) * (s2 <= k)

if((2 * t + 3) <= (j + 1) && k > 1) {

s21 <- (y[2 * t + 3] + s1)

s22 <- (y[2 * t + 2] + s2)

L2[t] <- (s21 + s22) * (s22 <= k)

}

if((2 * t + 5) <= (j + 1) && k > 2) {

s31 <- (y[2 * t + 5] + s21)

s32 <- (y[2 * t + 4] + s22)

L3[t] <- (s31 + s32) * (s32 <= k)

}

if((2 * t + 7) <= (j + 1) && k > 3) {

s41 <- (y[2 * t + 7] + s31)

s42 <- (y[2 * t + 6] + s32)

L4[t] <- (s41 + s42) * (s42 <= k)

}

if((2 * t + 9) <= (j + 1) && k > 4) {

s51 <- (y[2 * t + 9] + s41)

s52 <- (y[2 * t + 8] + s42)

L5[t] <- (s51 + s52) * (s52 <= k)

}

temp<- c((y[2 * t - 1]), (y[2 * t + 1]), L1[t], L2[t], L3[

t], L4[t], L5[t])

L[1,t] <- max((y[2 * t - 1]), (y[2 * t + 1]), L1[t], L2[t], L3[

t], L4[t], L5[t])

L[2,t]<- which(L[1,t]==temp)[1]

t <- t + 1

}

tt <-which(L[1,]==max(L[1,]))

if(length(tt)==1){

if(L[2,tt]!=2){coord <-c(sum(y[1:(tt*2-2)])+1,sum(y[1:(tt*2-2)])+max(L[1,]))}

if(L[2,tt]==2){coord <- c(sum(y[1:(tt*2)])+1,sum(y[1:(tt*2)])+max(L[1,])) }

}

if(length(tt)>=2){

for(jj in 1:(length(tt)-1)){

ttt<- ifelse(L[,tt[jj+1]][2]-L[,tt[jj]][2]==-1&tt[jj+1]-tt[jj]==1,tt[jj],tt[jj+1] )}

if(L[2,ttt]!=2){coord <-c(sum(y[1:(ttt*2-2)])+1,sum(y[1:(ttt*2-2)])+max(L[1,]))}

if(L[2,ttt]==2){coord <-c(sum(y[1:(ttt*2)])+1,sum(y[1:(ttt*2)])+max(L[1,])) }}

}

if(a[1] == 0) {

if((2 * t + 2) > (j + 1)) {

L <- y[2]

}

while((2 * t + 2) <= (j + 1)) {

s1 <- y[2 * t] + y[2 * t + 2]

s2 <- y[2 * t + 1]

L1[t] <- (s1 + s2) * (s2 <= k)

if((2 * t + 4) <= (j + 1) && k > 1) {

s21 <- (y[2 * t + 4] + s1)

s22 <- (y[2 * t + 3] + s2)

L2[t] <- (s21 + s22) * (s22 <= k)

}

if((2 * t + 6) <= (j + 1) && k > 2) {

s31 <- (y[2 * t + 6] + s21)

s32 <- (y[2 * t + 5] + s22)

L3[t] <- (s31 + s32) * (s32 <= k)

}

if((2 * t + 8) <= (j + 1) && k > 3) {

s41 <- (y[2 * t + 8] + s31)

s42 <- (y[2 * t + 7] + s32)

L4[t] <- (s41 + s42) * (s42 <= k)

}

if((2 * t + 10) <= (j + 1) && k > 4) {

s51 <- (y[2 * t + 10] + s41)

s52 <- (y[2 * t + 9] + s42)

L5[t] <- (s51 + s52) * (s52 <= k)

}

tt <-which(L[1,]==max(L[1,]))

temp<- c((y[2 * t]), (y[2 * t + 2]), L1[t], L2[t], L3[t], L4[

t], L5[t])

L[1,t] <- max((y[2 * t]), (y[2 * t + 2]), L1[t], L2[t], L3[t], L4[

t], L5[t])

L[2,t]<- which(L[1,t]==temp)[1]

t <- t + 1

}

tt <-which(L[1,]==max(L[1,]))

if(length(tt)==1){

if(L[2,tt]!=2){coord <-c(sum(y[1:(tt*2-1)])+1,sum(y[1:(tt*2-1)])+max(L[1,]))}

if(L[2,tt]==2){coord <- c(sum(y[1:(tt*2+1)])+1,sum(y[1:(tt*2+1)])+max(L[1,])) }

}

if(length(tt)>=2){

for(jj in 1:(length(tt)-1)){

ttt<- ifelse(L[,tt[jj+1]][2]-L[,tt[jj]][2]==-1&tt[jj+1]-tt[jj]==1,jj+1,jj )

}

if(L[2,ttt]!=2){coord <-c(sum(y[1:(ttt*2-1)])+1,sum(y[1:(ttt*2-1)])+max(L[1,]))}

if(L[2,ttt]==2){coord <- c(sum(y[1:(ttt*2+1)])+1,sum(y[1:(ttt*2+1)])+max(L[1,])) }

}

}

}

#result<-list(y=y,a=a,L1=L1,L2=L2,L3=L3,L4=L4,L5=L5,L=L)

#the max length of well-matching (1) run, (allowing k mismatches) in binary sequence

d <- max(L)

return(d,coord)

}

######################### subroutine 3 ###############################

Lk.0<-function(ps, pss, pff, n, d)

{

#this program compute the probability P(L0>=d)#

#L0 is the perfect matching run within a sequence of length n#

# ps is the initial prob. for "s", and pss is the transition prob of 1 given 1#

ad <- matrix(1, d, 1)

b <- diag(1, nrow=d, ncol = d)

c <- matrix(0, 1, d)

p0 <- cbind(1, c)

p0 <- cbind(p0, 0)

U <- rbind(ad, 0)

U <- rbind(1, U)

c <- cbind(c, 1)

M <- cbind((1 - pss) * ad, pss * b)

M <- rbind(M, c)

M <- cbind(0, M)

M <- rbind(0, M)

M[1, 2] <- 1 - ps

M[1, 3] <- ps

M[2, 2] <- pff

M[2, 3] <- 1 - pff

Mn <- M %*% M

for(i in 3:n) {

Mn <- Mn %*% M

}

1 - p0 %*% Mn %*% U

}

######################### subroutine 4 ###############################

Lk.1<-function(ps, pss, pff, n, d)

{

#this program compute the probability P(N(n,d)=0)#

#i.e., the number of non-overlapping type 1 runs having at most 1 type 0 #

#under given initial ps and pf with length d is zero.d=#1+#0#

NN <- (((1 + d) * d)/2) + 1

pf <- 1 - ps

psf <- 1 - pss

pfs <- 1 - pff

c0 <- matrix(0, 1, NN - 1)

p0 <- cbind(1, c0)

c1 <- matrix(1, NN - 1, 1)

U <- rbind(c1, 0)

M <- matrix(0, NN, NN)

for(ir in 0:(d - 1)) {

for(jr in 0:ir) {

for(ic in 0:(d - 1)) {

for(jc in 0:ic) {

rid <- (((ir + 1) * ir)/2) + jr + 1

cid <- (((ic + 1) * ic)/2) + jc + 1

if(ir > 0 && jr == 0 && ic == ir + 1 && jc == 0) {

M[rid, cid] <- pss

}

if(jr > 1 && ic == ir + 1 && jc == jr + 1) {

M[rid, cid] <- pss

}

if(ir > 0 && ic == ir + 1 && jr == 0 && jc == 1) {

M[rid, cid] <- psf

}

if(jr > 0 && ic == jr && jc == 1) {

M[rid, cid] <- psf

}

if(ir > 0 && jr == 1 && ic == 1 && jc == 1) {

M[rid, cid] <- pff

}

if(ir > 0 && jr == 1 && ic == ir + 1 && jc == 2) {

M[rid, cid] <- pfs

}

if(ir == 0 && jr == 0 && ic == 1 && jc == 0) {

M[rid, cid] <- ps

}

if(ir == 0 && jr == 0 && ic == 1 && jc == 1) {

M[rid, cid] <- pf

}

}

}

}

}

for(i in 1:NN) {

M[i, NN] <- 1 - sum(M[i, ])

}

Mn <- M %*% M

for(i in 3:n) {

Mn <- Mn %*% M

}

1 - p0 %*% Mn %*% U

}

######################### subroutine 5 ###############################

Lk.2<-function(ps, pss, pff, n, d)

{

#this program compute the probability P(L2>=d)#

#L2 is the 2-interrupted run within a sequence of length n#

# ps is the initial prob. for "s", and pss is the transition prob of 1 given 1#

NN <- (2 * d^3 + 10 * d)/12 + 1

pf <- 1 - ps

psf <- 1 - pss

pfs <- 1 - pff

c0 <- matrix(0, 1, NN - 1)

p0 <- cbind(1, c0)

c1 <- matrix(1, NN - 1, 1)

U <- rbind(c1, 0)

M <- matrix(0, NN, NN)

for(ir in 0:(d - 1)) {

for(jr in 0:ir) {

for(kr in 0:ir) {

for(ic in 0:(d - 1)) {

for(jc in 0:ic) {

for(kc in 0:ic) {

rid <- ((2 * ir^3 + 10 * ir)/12 + ((jr -

1) * (2 * ir - jr + 2))/2 + kr -

jr + 2) * (kr > jr) * (jr > 0) +

((2 * ir^3 + 10 * ir)/12 + ((

(jr - 1) * (2 * ir - jr + 2))/

2 + 2) * (jr > 0)) * (kr == 0) +

1 * (kr == 0) * (jr == 0)

cid <- ((2 * ic^3 + 10 * ic)/12 + ((jc -

1) * (2 * ic - jc + 2))/2 + kc -

jc + 2) * (kc > jc) * (jc > 0) +

((2 * ic^3 + 10 * ic)/12 + ((

(jc - 1) * (2 * ic - jc + 2))/

2 + 2) * (jc > 0)) * (kc == 0) +

1 * (kc == 0) * (jc == 0)

if(ir > 0) {

if(jr > 1 && kr > jr && ic == ir +

1 && jc == jr + 1 && kc ==

kr + 1) {

M[rid, cid] <- pss

}

if(jr > 1 && kr == 0 && ic == ir +

1 && jc == jr + 1 && kc ==

0) {

M[rid, cid] <- pss

}

if(jr == 0 && kr == 0 && ic == ir +

1 && kc == 0 && jc == 0)

{

M[rid, cid] <- pss

}

if(jr == 0 && kr == 0 && ic == ir +

1 && jc == 1 && kc == 0)

{

M[rid, cid] <- psf

}

if(jr > 1 && kr == 0 && ic == ir +

1 && jc == 1 && kc == jr +

1) {

M[rid, cid] <- psf

}

if(jr > 1 && kr > jr && ic == kr &&

jc == 1 && kc == jr + 1)

{

M[rid, cid] <- psf

}

if(jr == 1 && kr == 0 && ic == ir +

1 && jc == 1 && kc == 2)

{

M[rid, cid] <- pff

}

if(jr == 1 && kr > jr + 1 && ic ==

kr && jc == 1 && kc == 2)

{

M[rid, cid] <- pff

}

if(jr == 1 && kr == 2 && ic == 2 &&

jc == 1 && kc == 2) {

M[rid, cid] <- pff

}

if(jr == 1 && kr == 0 && ic == ir +

1 && jc == 2 && kc == 0)

{

M[rid, cid] <- pfs

}

if(jr == 1 && kr > jr && ic == ir +

1 && jc == jr + 1 && kc ==

kr + 1) {

M[rid, cid] <- pfs

}

}

if(ir == 0 && jr == 0 && kr == 0 && ic ==

1 && jc == 0 && kc == 0) {

M[rid, cid] <- ps

}

if(ir == 0 && jr == 0 && kr == 0 && ic ==

1 && jc == 1 && kc == 0) {

M[rid, cid] <- pf

}

}

}

}

}

}

}

for(i in 1:NN) {

M[i, NN] <- 1 - sum(M[i, ])

}

Mn <- M %*% M

for(i in 3:n) {

Mn <- Mn %*% M

}

1 - p0 %*% Mn %*% U

}

######################### subroutine 6 ###############################

Lk.3<-function(ps, pss, pff, n,d)

{

#this program compute the probability P(N(n,d)=0)#

#i.e., the number of non-overlapping type 1 runs having at most 3 type 0#

#under given initial ps and pf with length d is zero.d=#1+#0#

NN<-(d^4-2*d^3+11*d^2+14*d+24)/24

pf<-1-ps

psf<-1-pss

pfs<-1-pff

c0<-matrix(0,1,NN-1)

p0<-cbind(1,c0)

c1<-matrix(1,NN-1,1)

U<-rbind(c1,0)

M<-matrix(0,NN,NN)

for(ir in 0:(d-1)){

for(jr in 0:ir){

for(kr in 0:ir){

for(lr in 0:ir){

for(ic in 0:(d-1)){

for(jc in 0:ic){

for(kc in 0:ic){

for(lc in 0:ic){

if((kr>0 && kr<=jr)||(lr>0 && lr<=kr) ||

(jr==0 && ( kr!=0||lr!=0))|| (kr==0&&lr!=0)){rid<-0}

else{ rid<-(ir^4-2*ir^3+11*ir^2+14*ir)/24+1+(jr-1+

(-6*ir^2-6*ir-6*ir*jr^2+6*ir^2*jr+12*ir*jr+2*jr^3-6*jr^2+4*jr)/12+1+

(((2*ir-jr-kr+2)*(kr-jr-1)/2)*(kr>jr)+1+(lr-kr)*(lr>kr))*(kr>1))*(jr>0)}

if((kc>0 && kc<=jc)||(lc>0 && lc<=kc) ||

(jc==0 && ( kc!=0||lc!=0))|| (kc==0&&lc!=0)){cid<-0}

else{ cid<-(ic^4-2*ic^3+11*ic^2+14*ic)/24+1+(jc-1+

(-6*ic^2-6*ic-6*ic*jc^2+6*ic^2*jc+12*ic*jc+2*jc^3-6*jc^2+4*jc)/12+1+

(((2*ic-jc-kc+2)*(kc-jc-1)/2)*(kc>jc)+1+(lc-kc)*(lc>kc))*(kc>1))*(jc>0)}

if(jr!=1 && ir>0){

if(ic==ir+1 && kr==0 && jr==0 && kc==0 && jc==0){M[rid,cid]<-pss}

if(ic==ir+1 && jc==(jr+1)*(jr>0)&&kc==(kr+1)*(kr>0)&&

lc==(lr+1)*(lr>0)){M[rid,cid]<-pss}}

if(jr==1 && jc==1){

if(kr==0 && lr==0 && lc==0 && kc==2 && jc==1 &&

ic==ir+1){M[rid,cid]<-pff}

if(ic==ir+1 && kc==2 && lc==kr+1 && lr==0 && kr>1){M[rid,cid]<-pff}

if(lr>kr && kr>jr && ic==lr && kc==jr+1 && lc==kr+1){M[rid,cid]<-pff}}

if(jr==1){

if(kr==0 && lr==0 && ic==ir+1 && jc==2 && kc==0 &&

lc==0){M[rid,cid]<-pfs}

if(jc==2 && kc==kr+1 && ic==ir+1 && lc==0 && lr==0){M[rid,cid]<-pfs}

if(ic==ir+1 && jc==jr+1 && kc==kr+1 && lc==lr+1 && lr>kr&& kr>jr)

{M[rid,cid]<-pfs}}

if(ir>0 && jr!=1&& jc==1){

if(ic==ir+1){

if( kr==0 && kc==0 && jr==0 && lr==0 && lc==0){M[rid,cid]<-psf}

if( jr>1 && kc==jr+1 && lr==0 &&lc==0&&kr==0){M[rid,cid]<-psf}

if( jr>1 && kr>jr && kc==jr+1 && lr==0 && lc==kr+1)

{M[rid,cid]<-psf}}

if(ic==lr && jc==1 && kc==jr+1 && lc==kr+1&& jr>1&&

kr>jr && lr>kr ){M[rid,cid]<-psf}}

if(ir==0 && jr==0 && kr==0 && lr==0 && ic==1 && jc==0 && kc==0 && lc==0){M[rid,cid]<-ps}

if(ir==0 && jr==0 && kr==0 && lr==0 && ic==1 && jc==1 && kc==0 && lc==0){M[rid,cid]<-pf}

}}}}}}}}

for(i in 1:NN) { M[i,NN]<-1-sum(M[i,]) }

Mn<-M%*%M

for (i in 3:n) {Mn<-Mn%*%M }

1-p0%*%Mn%*%U

}

######################### subroutine 7 ###############################

Lk.waterman<-function(a , k)

{

#this program compute the probability of k-zero-run, P(Lk>=d),

# in a iid Bernoulli seq with P(1)=ps,

# where n is the length of seq.,

# d is the length of Lk, and k is the num. of mismached.

# output 3 values: est. of the prob, and its lower, upper bounds

n <- length(a)

d <- Lk.length(a, k)$d

ps <- sum(a)/n

s <- d - k

EEW <- (n * (s/d - ps) * dbinom(s, d, ps)) * (-1)

EEW <- exp(EEW)

erro <- 7 * d * dbinom(s, d, ps) + (1 - pbinom(s, d, ps))

upb <- EEW + erro

lob <- EEW - erro

bd <- c(EEW, upb, lob)

bd <- 1 - bd

#if(bd[2] > 1) bd[2] <- 1

#if(bd[3] < 0) bd[3] <- 0

if(bd[1] < 0) bd[1] <- 0

if(bd[1] > 1) bd[1] <- 1

bd[1]

}
