# Supplementary material for: Using the longest significance run to estimate region-specific p-values in genetic association mapping studies
Source: BMC Bioinformatics. 2008 May 27;9:246. doi: 10.1186/1471-2105-9-246 (PMC2430975; doi:10.1186/1471-2105-9-246)
Supplement: Additional file 2 — The details of the markers in the 0–1 sequence. The marker position and marker name of the 0–1 sequence. [file 1471-2105-9-246-S2.doc]

### Additional file 2 – The details of the markers in the 0-1 sequence.

|  | **Marker** | **Position** |  | **Marker** | **Position** |  | **Marker** | **Position** |
| --- | --- | --- | --- | --- | --- | --- | --- | --- |
| 1 | 416WTC84P | 30876 | 31 | 543WTC123P | 262259 | 61 | 317WTC65P | 332117 |
| 2 | 416WTC98P | 45993 | 32 | 543WTC124P | 262881 | 62 | MX5301 | 334008 |
| 3 | 416WTC53P | 55432 | 33 | 543WTC125P | 265108 | 63 | MX5302 | 334017 |
| 4 | 69WTC36P | 67935 | 34 | 543WTC24P | 266492 | 64 | 317WTC69P | 341145 |
| 5 | 69WTC1P | 68558 | 35 | 543WTC46WP | 267295 | 65 | 317WTC70P | 341403 |
| 6 | 416WTC89P | 103182 | 36 | 543WTC45WP | 267348 | 66 | 317WTC74P | 342981 |
| 7 | 69WTC12P | 129002 | 37 | 543WTC44WP | 267449 | 67 | 317WTC75P | 344136 |
| 8 | 416WTC96P | 145407 | 38 | 543WTC110P | 267901 | 68 | 317WTC9P | 347028 |
| 9 | 543WTC21P | 191388 | 39 | DP1020 | 268075 | 69 | 317WTC71P | 351373 |
| 10 | 543WTC100P | 208834 | 40 | 543WTC42P | 276913 | 70 | 317WTC72P | 354755 |
| 11 | 543WTC49WP | 212826 | 41 | 543WTC43P | 277495 | 71 | MX6392 | 359096 |
| 12 | 543WTC41P | 245532 | 42 | 543WTC81P | 282621 | 72 | 317WTC76P | 359224 |
| 13 | 543WTC31P | 247247 | 43 | 16WTC126P | 296131 | 73 | MX6601 | 370001 |
| 14 | 543WTC33P | 247709 | 44 | 16WTC105P | 299520 | 74 | 317WTC7P | 368313 |
| 15 | 543WTC34P | 247827 | 45 | 16WTC106P | 301602 | 75 | 317WTC30P | 380193 |
| 16 | DP1011 | 247839 | 46 | 16WTC104P | 305609 | 76 | 317WTC14P | 389388 |
| 17 | 543WTC112P | 249018 | 47 | 317WTC61P | 317735 | 77 | 317WTC16P | 390042 |
| 18 | 543WTC114P | 250618 | 48 | 317WTC62P | 317891 | 78 | 317WTC28P | 407126 |
| 19 | 543WTC115P | 250846 | 49 | 317WTC59P | 318524 | 79 | 317WTC82P | 415357 |
| 20 | 543WTC90P | 252117 | 50 | 317WTC66P | 319819 |  |  |  |
| 21 | 543WTC91P | 252279 | 51 | 317WTC67P | 320386 |  |  |  |
| 22 | DP1037 | 256378 | 52 | 317WTC68P | 321029 |  |  |  |
| 23 | DP1039 | 256845 | 53 | 317WTC55P | 322250 |  |  |  |
| 24 | DP1041 | 257046 | 54 | 317WTC56P | 322328 |  |  |  |
| 25 | 543WTC121P | 257959 | 55 | 317WTC57P | 323846 |  |  |  |
| 26 | DP1043 | 258094 | 56 | 317WTC108P | 324451 |  |  |  |
| 27 | 543WTC122P | 259007 | 57 | 317WTC127P | 325873 |  |  |  |
| 28 | D2S308 | 261056 | 58 | 317WTC60P | 326869 |  |  |  |
| 29 | 543WTC38P | 262047 | 59 | 317WTC58P | 329420 |  |  |  |
| 30 | 543WTC39P | 262128 | 60 | 317WTC64P | 332092 |  |  |  |
